# Supplementary material for: Epigenomic analysis of formalin-fixed paraffin-embedded samples by CUT&Tag
Source: Nat Commun. 2023 Sep 22;14:5930. doi: 10.1038/s41467-023-41666-z (PMC10516967; doi:10.1038/s41467-023-41666-z)
Supplement: Supplementary file 1 — Supplementary Information [file 41467_2023_41666_MOESM1_ESM.pdf]

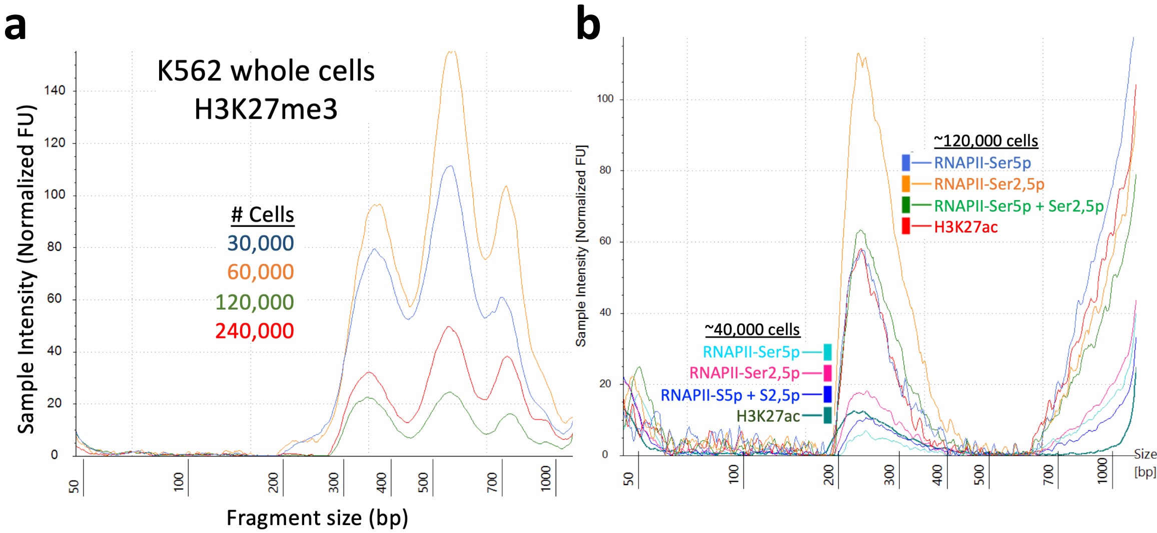

Supplementary Figure 1: Gel analysis of CUT&Tag and CUTAC libraries performed using Thermolabile Proteinase K post-tagmentation treatment. a) Representative Tapestation profiles for whole-cell CUT&Tag-direct. A log culture of K562 cells was supplemented with 10% DMSO, concentrated to 2 million cells/ml, aliquoted, slow-frozen in Mr. Frosty containers and stored at -80 °C. An aliquot was thawed and 15-60 µL was dispensed into PCR tubes for CUT&Tag-direct using an H3K27me3 antibody (CST cat. no. 9733). **b)** Tapestation profiles for FFPE CUTAC samples pre-incubated at 85 °C for 12 hr using four different antibodies. Each sample was divided 3/4-1/4 in the TAPS-wash before fragment release. Antibodies diluted 1:25 were RNAPII-Ser5p Cell Signaling Technology #13523, RNAPII-Ser2,5 Cell Signaling Technology #13546 and H3K27ac: Abcam #4729. A 10-micron section of a mouse brain tumor FFPE was deparaffinized using xylene. Note that both the CUTAC peaks the high-molecular weight smears scale with the amount of sample, likely representing ambient RNAs, which do not interfere with flow cell runs.

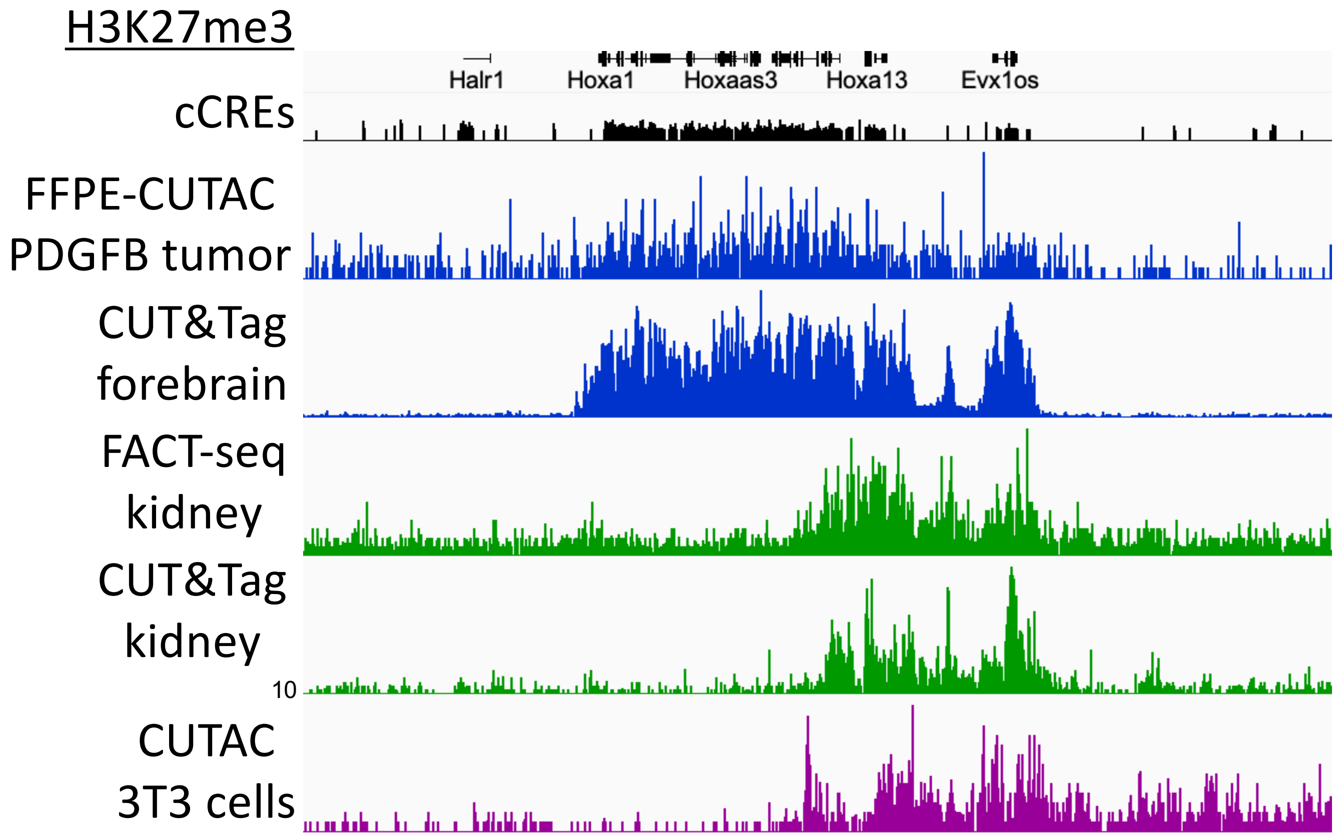

Supplementary Figure 2: Poor signal-to-noise for H3K27me3 FFPE-CUTAC. Comparison of H3K27me3 FFPE-CUTAC to H3K27me3 CUT&Tag (E12.5 forebrain GSM5904439). Adult kidney FACT-seq (GSM5530659) and CUT&Tag (GSM5530665) and 3T3 cell CUTAC are shown for reference.

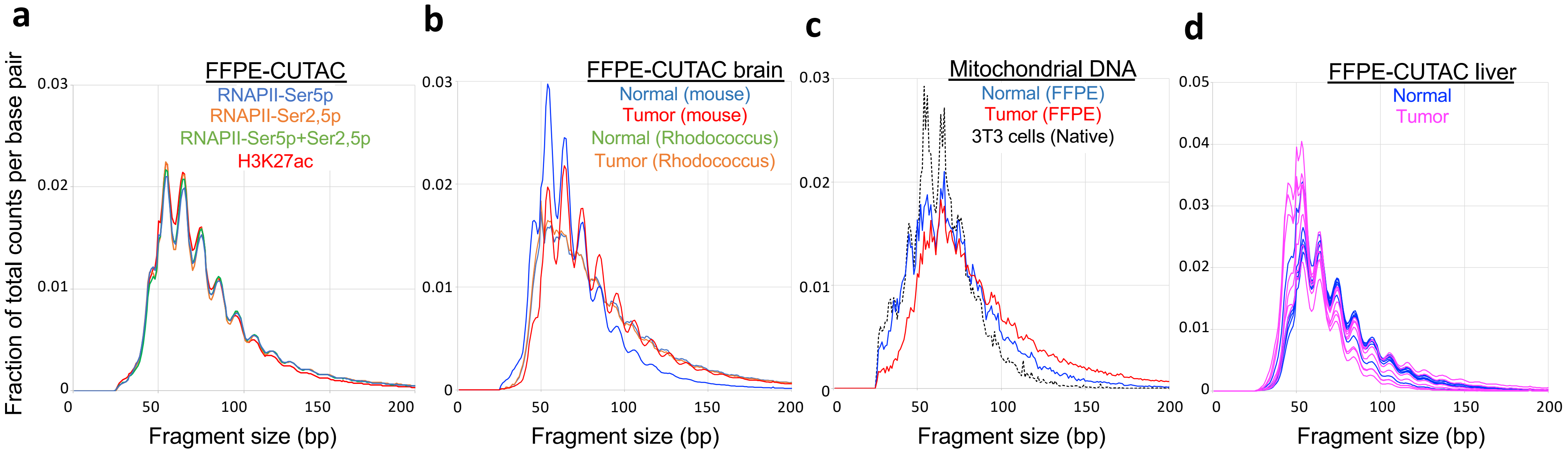

Supplementary Figure 3: Length distributions vary between tumor and normal. a) Average length distributions for three single antibodies (RNAPII-Ser5p: 15 samples; RNAPII-Ser2,5p: 15 samples; H3K27ac: 15 samples) and a 50:50 mixture of RNAPII-Ser5 and RNAPII-Ser2,5p: 14 samples. For each sample, mouse and *Rhodococcus* fragment lengths were divided by the total number of fragments before averaging. Lengths are plotted at single base-pair resolution. **b**) Average length distributions for the same samples as in (a) except grouped by cancer driver transgene (YAP1: 23 samples; PDGFB: 8 samples; RELA: 8 samples) and Normal brain: 20 samples. **c**) Same as (a) except for Mm10 ChrM (mitochondrial) fragments from the same FFPEs as used for panels e and f. The length distribution of Mm10 ChrM fragments from mouse 3T3 cells is plotted for reference. **d**) Same as (a) except that individual curves for liver tumors (magenta, 7 samples) and normal livers (blue, 6 samples) are superimposed.

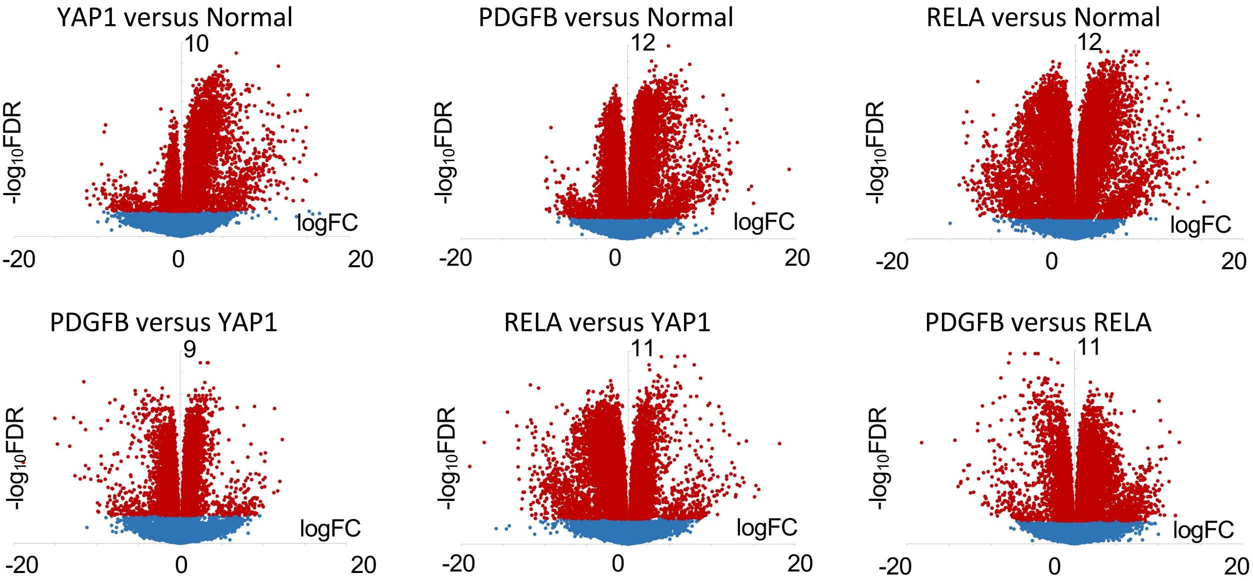

Supplementary Figure 4: Volcano plots of RNA-seq comparisons. YAP1: 3 replicates; PDGFB: 4 replicates; RELA: 4 replicates; Normal: 7 replicates.
