# Supplementary material for: Epigenomic analysis of formalin-fixed paraffin-embedded samples by CUT&Tag
Source: Nat Commun. 2023 Sep 22;14:5930. doi: 10.1038/s41467-023-41666-z (PMC10516967; doi:10.1038/s41467-023-41666-z)
Supplement: Supplementary file 3 — Description of Additional Supplementary Files Document [file 41467_2023_41666_MOESM3_ESM.pdf]

Description of Additional Supplementary Files

Supplementary Data 1: Mapping statistics and yields for FFPE-CUTAC bead-binding and on-slide protocols.

Supplementary Data 2: Summary of significant cCRE differences between FFPE-CUTAC datasets. (Related to Figure 4)**.** FDR = False Discovery Rate, which corrects for multiple comparisons, is based on average of normalized counts per base-pair in each cCRE. For each antibody or antibody mixture, replicates were down-sampled and equal numbers of fragments were merged for each genotype. Rank-ordered cCREs for each comparison based on summits are tabulated in Supplementary Table 3.

Supplementary Data 3_1: Top RNAPII-Ser5 up-regulated differences between RELA-driven Tumor and normal sections dissected from a single FFPE slide (Related to Figure 6c-d). FDR = False Discovery Rate, which corrects for multiple comparisons.

Supplementary Data 3_2: Top RNAPII-Ser5 down-regulated differences between RELA-driven Tumor and normal sections dissected from a single FFPE slide (Related to Figure 6c-d). FDR = False Discovery Rate, which corrects for multiple comparisons.

Supplementary Data 4_1: Top FFPE-CUTAC hits intersected with RNA-seq data from brain tumors (Related to Figure 8b). FDR = False Discovery Rate, which corrects for multiple comparisons.

Supplementary Data 4_2: Top FFPE-CUTAC hits intersected with RNA-seq data from brain tumors, rank-ordered (Related to Figure 8b). FDR = False Discovery Rate, which corrects for multiple comparisons.

Supplementary Data 5_1: Top hits for YAP1-versus-Normal FDR < 0.05 comparisons (Related to Figure 4d). FFPE-CUTAC replicates using RNAPII-Ser5p, RNAPII-Ser2,5p, RNAPII-Ser5p + RNAPII-Ser2,5p and H3K27ac from YAP1, PDGFB, RELA and Normal FFPEs were pooled for each antibody/genotype combination (4x4=16) and downsampled to the same level for each antibody or combination. Voom/Limma tumor-versus-normal differences were rank ordered by FDR < 0.05. FDR = False Discovery Rate, which corrects for multiple comparisons.

Supplementary Data 5_2: Top hits for PDGFB-versus-Normal FDR < 0.05 comparisons (Related to Figure 4d). FFPE-CUTAC replicates using RNAPII-Ser5p, RNAPII-Ser2,5p, RNAPII-Ser5p + RNAPII-Ser2,5p and H3K27ac from YAP1, PDGFB, RELA and Normal FFPEs were pooled for each antibody/genotype combination (4x4=16) and downsampled to the same level for each antibody or combination. Voom/Limma tumor-versus-normal differences were rank ordered by FDR < 0.05. FDR = False Discovery Rate, which corrects for multiple comparisons.

Supplementary Data 5_3: Top hits for RELA-versus-Normal FDR < 0.05 comparisons (Related to Figure 4d). FFPE-CUTAC replicates using RNAPII-Ser5p, RNAPII-Ser2,5p, RNAPII-Ser5p + RNAPII-Ser2,5p and H3K27ac from YAP1, PDGFB, RELA and Normal FFPEs were pooled for each antibody/genotype combination (4x4=16) and downsampled to the same level for each antibody or combination. Voom/Limma tumor-versus-normal differences were rank ordered by FDR < 0.05. FDR = False Discovery Rate, which corrects for multiple comparisons.

Supplementary Data 5_4: Top hits for YAP1-versus-PDGFB FDR < 0.05 comparisons (Related to Figure 4d). FFPE-CUTAC replicates using RNAPII-Ser5p, RNAPII-Ser2,5p, RNAPII-Ser5p + RNAPII-Ser2,5p and H3K27ac from YAP1, PDGFB, RELA and Normal FFPEs were pooled for each antibody/genotype combination (4x4=16) and downsampled to the same level for each antibody or combination. Voom/Limma tumor-versus-normal differences were rank ordered by FDR < 0.05. FDR = False Discovery Rate, which corrects for multiple comparisons.

Supplementary Data 5_5: Top hits for YAP1-versus-Normal FDR < 0.05 comparisons (Related to Figure 4d). FFPE-CUTAC replicates using RNAPII-Ser5p, RNAPII-Ser2,5p, RNAPII-Ser5p + RNAPII-Ser2,5p and H3K27ac from YAP1, PDGFB, RELA and Normal FFPEs were pooled for each antibody/genotype combination (4x4=16) and downsampled to the same level for each antibody or combination. Voom/Limma tumor-versus-normal differences were rank ordered by FDR < 0.05. FDR = False Discovery Rate, which corrects for multiple comparisons.

Supplementary Data 6_1: Top hits for RELA-versus-Normal FDR < 0.05 comparisons for on-slide FFPE-CUTAC (Related to Figure 6c-d). FDR = False Discovery Rate, which corrects for multiple comparisons.

Supplementary Data 6_2: Top hits for PDGFB-versus-Normal FDR < 0.05 comparisons for on-slide FFPE-CUTAC (Related to Figure 6c-d). FDR = False Discovery Rate, which corrects for multiple comparisons.

Supplementary Data 7: Top hits for Tumor-versus-Normal FDR < 0.05 comparisons for liver tumor-versus-normal (Related to Figure 7). FDR = False Discovery Rate, which corrects for multiple comparisons.

Supplementary Data 7: Top hits for Tumor-versus-Normal FDR < 0.05 comparisons for liver tumor-versus-normal (Related to Figure 7).

Supplementary Data 8_1: Final Library Structure

Supplementary Data 8_2: Custom Barcodes Adapter 1 (index i5) IDs: Buenrostro et al. 2015 https://doi.org/10.1038/nature14590

Supplementary Data 8_3: Custom Barcodes Adapter 2 (index i7) IDs: Buenrostro et al. 2015 https://doi.org/10.1038/nature14590
