# Supplementary material for: Epigenomic analysis of formalin-fixed paraffin-embedded samples by CUT&Tag
Source: Nat Commun. 2023 Sep 22;14:5930. doi: 10.1038/s41467-023-41666-z (PMC10516967; doi:10.1038/s41467-023-41666-z)
Supplement: Supplementary file 12 — Reporting Summary [file 41467_2023_41666_MOESM12_ESM.pdf]

Corresponding author(s): Steven Henikoff

Last updated by author(s): September 9, 2023

# Reporting Summary

Nature Portfolio wishes to improve the reproducibility of the work that we publish. This form provides structure for consistency and transparency in reporting. For further information on Nature Portfolio policies, see our Editorial Policies and the Editorial Policy Checklist.

## Statistics

For all statistical analyses, confirm that the following items are present in the figure legend, table legend, main text, or Methods section.

| n/a | Confirmed | |
|---|---|---|
| ☐ | ☒ | The exact sample size (*n*) for each experimental group/condition, given as a discrete number and unit of measurement |
| ☒ | ☐ | A statement on whether measurements were taken from distinct samples or whether the same sample was measured repeatedly |
| ☐ | ☒ | The statistical test(s) used AND whether they are one- or two-sided<br>*Only common tests should be described solely by name; describe more complex techniques in the Methods section.* |
| ☒ | ☐ | A description of all covariates tested |
| ☒ | ☐ | A description of any assumptions or corrections, such as tests of normality and adjustment for multiple comparisons |
| ☐ | ☒ | A full description of the statistical parameters including central tendency (e.g. means) or other basic estimates (e.g. regression coefficient) AND variation (e.g. standard deviation) or associated estimates of uncertainty (e.g. confidence intervals) |
| ☐ | ☒ | For null hypothesis testing, the test statistic (e.g. *F*, *t*, *r*) with confidence intervals, effect sizes, degrees of freedom and *P* value noted<br>*Give P values as exact values whenever suitable.* |
| ☒ | ☐ | For Bayesian analysis, information on the choice of priors and Markov chain Monte Carlo settings |
| ☒ | ☐ | For hierarchical and complex designs, identification of the appropriate level for tests and full reporting of outcomes |
| ☒ | ☐ | Estimates of effect sizes (e.g. Cohen's *d*, Pearson's *r*), indicating how they were calculated |

*Our web collection on statistics for biologists contains articles on many of the points above.*

## Software and code

Policy information about availability of computer code

| | |
|---|---|
| Data collection | The size distributions and molar concentration of libraries were determined using an Agilent 4200 TapeStation. Up to 48 barcoded CUT&RUN libraries or 96 barcoded CUT&Tag libraries were pooled at approximately equimolar concentration for sequencing. Paired-end 50x50 bp sequencing on the Illumina NextSeq 2000 platform was performed by the Fred Hutchinson Cancer Research Center Genomics Shared Resources. This yielded 1-20 million reads per antibody. Adapters were clipped by cutadapt version 4.1 (51) with parameters<br>-j 8 --nextseq-trim 20 -m 20 -a AGATCGGAAGAGCACACGTCTGAACTCCAGTCA -A AGATCGGAAGAGCGTCGTGTAGGGAAAGAGTGT -Z<br>Clipped reads were aligned by Bowtie2 version 2.4.4 to the Mus musculus mm10 and Homo sapiens hg19 reference sequences from UCSC and to the Rhodococcus erythropolis complete genome (NZ_CP007255.1) from NCBI with parameters<br>--very-sensitive-local --soft-clipped-unmapped-tlen --dovetail --no-mixed --no-discordant -q --phred33 -I 10 -X 1000 |
| Data analysis | Bedtools; Voom/Limma option on the Degust server https://degust.erc.monash.edu/degust/. Picard Tools: MarkDuplicates http://broadinstitute.github.io/picard/ |

For manuscripts utilizing custom algorithms or software that are central to the research but not yet described in published literature, software must be made available to editors and reviewers. We strongly encourage code deposition in a community repository (e.g. GitHub). See the Nature Portfolio guidelines for submitting code & software for further information.

## Data

Policy information about availability of data

All manuscripts must include a data availability statement. This statement should provide the following information, where applicable:

- Accession codes, unique identifiers, or web links for publicly available datasets
- A description of any restrictions on data availability
- For clinical datasets or third party data, please ensure that the statement adheres to our policy

All primary sequencing data have been deposited as paried-end fastq files in Gene Expression Omnibus under the accession code GSE224579.

## Research involving human participants, their data, or biological material

Policy information about studies with human participants or human data. See also policy information about sex, gender (identity/presentation), and sexual orientation and race, ethnicity and racism.

| | |
|---|---|
| Reporting on sex and gender | Not applicable |
| Reporting on race, ethnicity, or other socially relevant groupings | Not applicable |
| Population characteristics | Not applicable |
| Recruitment | Not applicable |
| Ethics oversight | Not applicable |

Note that full information on the approval of the study protocol must also be provided in the manuscript.

# Field-specific reporting

Please select the one below that is the best fit for your research. If you are not sure, read the appropriate sections before making your selection.

☒ Life sciences ☐ Behavioural & social sciences ☐ Ecological, evolutionary & environmental sciences

For a reference copy of the document with all sections, see nature.com/documents/nr-reporting-summary-flat.pdf

# Life sciences study design

All studies must disclose on these points even when the disclosure is negative.

| | |
|---|---|
| Sample size | One 5 or 10 micron section from an FFPE block or a dissected portion of an FFPE for each 5- or 10-micron specimen used in Voom/Limma analysis. Sample sizes were not determined in advance but rather by the amount of tissue on a section divided by the number of samples from the section. |
| Data exclusions | Sequencing reads mapping to the mitochondrial genome were removed from all datasets. This was pre-established and is standard practice in the field. The purpose of this study was to perform comparative analysis of chromatin profiles from the nuclear genome and this can be confounded by variable read numbers from the mitochondrial genome. |
| Replication | For Voom/Limma analysis at least 2 replicates were used. Replicates were successful. |
| Randomization | n/a. The data and analysis for this study is objective and not prone to influence by the researchers bias. |
| Blinding | n/a. The data and analysis for this study is objective and not prone to influence by researchers bias. |

# Reporting for specific materials, systems and methods

We require information from authors about some types of materials, experimental systems and methods used in many studies. Here, indicate whether each material, system or method listed is relevant to your study. If you are not sure if a list item applies to your research, read the appropriate section before selecting a response.

## Materials & experimental systems

| n/a | Involved in the study |
| --- | --- |
| ☐ | ☒ Antibodies |
| ☐ | ☒ Eukaryotic cell lines |
| ☒ | ☐ Palaeontology and archaeology |
| ☐ | ☒ Animals and other organisms |
| ☒ | ☐ Clinical data |
| ☒ | ☐ Dual use research of concern |
| ☒ | ☐ Plants |

## Methods

| n/a | Involved in the study |
| --- | --- |
| ☐ | ☒ ChIP-seq |
| ☒ | ☐ Flow cytometry |
| ☒ | ☐ MRI-based neuroimaging |

# Antibodies

Antibodies used

Primary antibodies: H3K4me3: Active Motif cat. no. 39159, lot no. 18122006; H3K27ac: Abcam cat. no. ab4729, lot no. 1033973; RNAPII-Ser5p: Cell Signaling Technologies cat. no. 13523, lot 3; RNAPII-Ser2,5p: Cell Signaling Technologies cat. no. 13546, lot 1; H3K27me3: Cell Signaling Technologies cat. no. 9733, lot 19; H3K4me2: Epicypher cat. no. 13-0027, lot 21090003-01; H3K36me3: Thermo cat. no. MAS-24687, lot VE2997961. Secondary antibody: Guinea pig α-rabbit antibody (Antibodies online cat. no. ABIN101961, lot 46671).

Validation

All antibodies used in this study were confirmed by the manufacturer to recognize the epitope as stated on the manufactures website. Links to manufacturer's validations: H3K4me3: https://www.activemotif.com/catalog/details/39159/histone-h3-trimethyl-lys4-antibody-pab; H3K27ac: https://www.abcam.com/products/primary-antibodies/histone-h3-acetyl-k27-antibody-chip-grade-ab4729.html#lb RNAPII-Ser5p: Cell Signaling Technologies cat. no. 13523, lot 3; RNAPII-Ser2,5p: Cell Signaling Technologies cat. no. 13546: https://www.cellsignal.com/products/primary-antibodies/phospho-rpb1-ctd-ser2-ser5-d1g3k-rabbit-mab/13546?_requestid=265202; H3K27me3: https://www.cellsignal.com/products/primary-antibodies/tri-methyl-histone-h3-lys27-c36b11-rabbit-mab/9733; H3K4me2: https://www.epicypher.com/content/documents/tds/13-0027.pdf; H3K36me3: https://www.thermofisher.com/antibody/product/H3K36me3-Antibody-clone-RM155-Recombinant-Monoclonal/MA5-24687.

# Eukaryotic cell lines

Policy information about cell lines and Sex and Gender in Research

Cell line source(s)

Human K562 cells were purchased from ATCC (Manassas, VA, Cat# CCL-243),
H1 hESCs were obtained from WiCell (Cat# WA01-lot# WB35186),
Mouse 3T3 cells were obtained from the Sarthy lab at Seattle Children's Hospital.

Authentication

All the cell lines used in this study are regularly submitted for karyotyping by the Fred Hutchinson Cancer Center Core Facilities.

Mycoplasma contamination

All cell lines were confirmed as mycoplasma negative on a tri-monthly basis.

Commonly misidentified lines
(See ICLAC register)

No commonly misidentified lines were used in this study.

# Animals and other research organisms

Policy information about studies involving animals; ARRIVE guidelines recommended for reporting animal research, and Sex and Gender in Research

Laboratory animals

We used both male and female mice from Jackson lab mouse strain 3529: https://www.jax.org/strain/003529 (FVB/N;C57BL/6;129/Sv). Nestin (N)/tv-a Cdkn2a null pups (P0-P1; male and female) or adults (5-7 week old, male and female) were injected intracranially with either RCAS-PDGFB, RCAS-YAP1-FAM118B, or RCAS-ZFTA-RELA-expressing DF-1 cells and monitored daily for tumor related symptoms for the duration of the experiment. Upon weaning (~P21), mice were housed with same-sex littermates, with no more than 5 per cage and given access to food/water ad libitum. All animal experiments were approved by and conducted in accordance with the Institutional Animal Care and Use Committee of Fred Hutchinson Cancer Center (Protocol #50842: Tva-derived transgenic mouse model for studying brain tumors).

Wild animals

No wild animals were used in this study.

Reporting on sex

We used both male and female mice.

Field-collected samples

No field-collected samples were used in this study.

Ethics oversight

This research was approved by the Fred Hutch Institutional Animal Care and Use Committee (Protocol # 50842) and complies with all required ethical regulations.

Note that full information on the approval of the study protocol must also be provided in the manuscript.

# Plants

| | |
|---|---|
| Seed stocks | Not applicable |
| Novel plant genotypes | Not applicable |
| Authentication | Not applicable |

# ChIP-seq

## Data deposition

☒ Confirm that both raw and final processed data have been deposited in a public database such as GEO.

☒ Confirm that you have deposited or provided access to graph files (e.g. BED files) for the called peaks.

| | |
|---|---|
| Data access links<br>*May remain private before publication.* | *For "Initial submission" or "Revised version" documents, provide reviewer access links. For your "Final submission" document, provide a link to the deposited data.* |

Files in database submission

| | |
|---|---|
| GSE235876 | Epigenomic analysis of Formalin-Fixed Paraffin-Embedded samples by CUT&Tag |
| GSM7511134 | 3T3_H3K27me3_3T3_nuclei_(230417_SH_Mm_K27m3_3T3_0405) |
| GSM7511135 | 3T3_H3K36me3_3T3_nuclei_(230417_SH_Mm_K36m3_3T3_0405) |
| GSM7511136 | 3T3_H3K4me2_3T3_nuclei_(230417_SH_Mm_K4m2_3T3_0405) |
| GSM7511137 | 3T3_H3K4me3_3T3_nuclei_(230417_SH_Mm_K4m3_3T3_0405) |
| GSM7511138 | 3T3_RNAPol2-Ser5_3T3_nuclei_(230417_SH_Mm_P2S5_3T3_0405) |
| GSM7511139 | H3K27ac_Normal_brain_1_(230404_SH_Mm_K27ac_N1_0330) |
| GSM7511140 | H3K27ac_Normal_brain_1_high_(230512_SH_Mm_K27ac_N1H_0506) |
| GSM7511141 | H3K27ac_Normal_brain_1_low_(230512_SH_Mm_K27ac_N1l_0506) |
| GSM7511142 | H3K27ac_Normal_brain_2_(230410_SH_Mm_K27ac_N21_0401) |
| GSM7511143 | H3K27ac_Normal_brain_2_high_(230512_SH_Mm_K27ac_N2H_0506) |
| GSM7511144 | H3K27ac_Normal_brain_2_low_(230512_SH_Mm_K27ac_N2l_0506) |
| GSM7511145 | H3K27ac_Normal_brain_3_(230404_SH_Mm_K27ac_N3_0330) |
| GSM7511146 | H3K27ac_Normal_brain_3_high_(230512_SH_Mm_K27ac_N3H_0506) |
| GSM7511147 | H3K27ac_Normal_brain_3_low_(230512_SH_Mm_K27ac_N3l_0506) |
| GSM7511148 | H3K27ac_Normal_brain_mineraloil_(230428_SH_Mm_K27ac_N80m_0425) |
| GSM7511149 | H3K27ac_PDGFB_3_(230404_SH_Mm_K27ac_P_0330) |
| GSM7511150 | H3K27ac_PDGFB_SDS_(230321_SH_Mm_K27ac_PS_0319) |
| GSM7511151 | H3K27ac_PDGFB_Triton_(230321_SH_Mm_K27ac_PT_0319) |
| GSM7511152 | H3K27ac_PDGFB_high_(230512_SH_Mm_K27ac_PH_0506) |
| GSM7511153 | H3K27ac_PDGFB_low_(230512_SH_Mm_K27ac_Pl_0506) |
| GSM7511154 | H3K27ac_RELA_1_(230410_SH_Mm_K27ac_R1_0401) |
| GSM7511155 | H3K27ac_RELA_2_(230410_SH_Mm_K27ac_R2_0401) |
| GSM7511156 | H3K27ac_RELA_3_(230404_SH_Mm_K27ac_R_0330) |
| GSM7511157 | H3K27ac_RELA_4_(230410_SH_Mm_K27ac_R4_0401) |
| GSM7511158 | H3K27ac_RELA_8_(230410_SH_Mm_K27ac_R8_0401) |
| GSM7511159 | H3K27ac_RELA_high_(230512_SH_Mm_K27ac_RH_0506) |
| GSM7511160 | H3K27ac_RELA_low_(230512_SH_Mm_K27ac_Rl_0506) |
| GSM7511161 | H3K27ac_YAP1_1_(230404_SH_Mm_K27ac_Y1_0330) |
| GSM7511162 | H3K27ac_YAP1_11_(230410_SH_Mm_K27ac_Y11_0401) |
| GSM7511163 | H3K27ac_YAP1_12_(230410_SH_Mm_K27ac_Y12_0401) |
| GSM7511164 | H3K27ac_YAP1_14_(230410_SH_Mm_K27ac_Y14_0401) |
| GSM7511165 | H3K27ac_YAP1_18_(230410_SH_Mm_K27ac_Y18_0401) |
| GSM7511166 | H3K27ac_YAP1_2_(230404_SH_Mm_K27ac_Y2_0330) |
| GSM7511167 | H3K27ac_YAP1_3_(230404_SH_Mm_K27ac_Y3_0330) |
| GSM7511168 | H3K27ac_YAP1_high_(230512_SH_Mm_K27ac_Y1H_0506) |
| GSM7511169 | H3K27ac_YAP1_low_(230512_SH_Mm_K27ac_Y1l_0506) |
| GSM7511170 | H3K27ac_YAP2_high_(230512_SH_Mm_K27ac_Y2H_0506) |
| GSM7511171 | H3K27ac_YAP2_low_(230512_SH_Mm_K27ac_Y2l_0506) |
| GSM7511172 | H3K27ac_YAP3_high_(230512_SH_Mm_K27ac_Y3H_0506) |
| GSM7511173 | K27ac_PDGFB_1_(230301_SH_Mm_KacL_PD-B5_0219) |
| GSM7511174 | K27ac_PDGFB_2_(230308_SH_Mm_K27ac_Rx8_0225) |
| GSM7511175 | K27ac_RELA_1_(230308_SH_Mm_K27ac_Px8_0225) |
| GSM7511176 | RNAPol2-Ser25_Normal_brain_1_high_(230512_SH_Mm_P2S25_N1H_0506) |
| GSM7511177 | RNAPol2-Ser25_Normal_brain_1_low_(230512_SH_Mm_P2S25_N1l_0506) |
| GSM7511178 | RNAPol2-Ser25_Normal_brain_2_high_(230512_SH_Mm_P2S25_N2H_0506) |
| GSM7511179 | RNAPol2-Ser25_Normal_brain_3_high_(230512_SH_Mm_P2S25_N3H_0506) |
| GSM7511180 | RNAPol2-Ser25_Normal_brain_3_low_(230512_SH_Mm_P2S25_N3l_0506) |
| GSM7511181 | RNAPol2-Ser25_PDGFB_1_(230308_SH_Mm_P2S25_Px8_0225) |
| GSM7511182 | RNAPol2-Ser25_PDGFB_6_(230428_SH_Mm_P2S25_P85_0422) |
| GSM7511183 | RNAPol2-Ser25_PDGFB_high_(230512_SH_Mm_P2S25_PH_0506) |
| GSM7511184 | RNAPol2-Ser25_PDGFB_low_(230512_SH_Mm_P2S25_Pl_0506) |
| GSM7511185 | RNAPol2-Ser25_RELA_1_(230308_SH_Mm_P2S25_Rx8_0225) |
| GSM7511186 | RNAPol2-Ser25_RELA_5_(230417_SH_Mm_P2S25_RLo_0408) |

| | |
|---|---|
| GSM7511187 | RNAPol2-Ser25_RELA_high_(230512_SH_Mm_P2S25_RH_0506) |
| GSM7511188 | RNAPol2-Ser25_RELA_low_(230512_SH_Mm_P2S25_Rl_0506) |
| GSM7511189 | RNAPol2-Ser25_YAP1_high_(230512_SH_Mm_P2S25_Y1H_0506) |
| GSM7511190 | RNAPol2-Ser25_YAP1_low_(230512_SH_Mm_P2S25_Y1l_0506) |
| GSM7511191 | RNAPol2-Ser25_YAP2_high_(230512_SH_Mm_P2S25_Y2H_0506) |
| GSM7511192 | RNAPol2-Ser25_YAP2_low_(230512_SH_Mm_P2S25_Y2l_0506) |
| GSM7511193 | RNAPol2-Ser25_YAP3_high_(230512_SH_Mm_P2S25_Y3H_0506) |
| GSM7511194 | RNAPol2-Ser25_YAP3_low_(230512_SH_Mm_P2S25_Y3l_0506) |
| GSM7511195 | RNAPol2-Ser525_Normal_brain_1_high_(230512_SH_Mm_P2S525_N1H_0506) |
| GSM7511196 | RNAPol2-Ser525_Normal_brain_1_low_(230512_SH_Mm_P2S525_N1l_0506) |
| GSM7511197 | RNAPol2-Ser525_Normal_brain_2_high_(230512_SH_Mm_P2S525_N2H_0506) |
| GSM7511198 | RNAPol2-Ser525_Normal_brain_3_low_(230512_SH_Mm_P2S525_N3l_0506) |
| GSM7511199 | RNAPol2-Ser525_PDGFB_high_(230512_SH_Mm_P2S525_PH_0506) |
| GSM7511200 | RNAPol2-Ser525_PDGFB_low_(230512_SH_Mm_P2S525_Pl_0506) |
| GSM7511201 | RNAPol2-Ser525_RELA_high_(230512_SH_Mm_P2S525_RH_0506) |
| GSM7511202 | RNAPol2-Ser525_RELA_low_(230512_SH_Mm_P2S525_Rl_0506) |
| GSM7511203 | RNAPol2-Ser525_YAP1_(230512_SH_Mm_P2S525_Y1_0506) |
| GSM7511204 | RNAPol2-Ser525_YAP1_high_(230512_SH_Mm_P2S525_Y1H_0506) |
| GSM7511205 | RNAPol2-Ser525_YAP2_high_(230512_SH_Mm_P2S525_Y2H_0506) |
| GSM7511206 | RNAPol2-Ser525_YAP2_low_(230512_SH_Mm_P2S525_Y2l_0506) |
| GSM7511207 | RNAPol2-Ser525_YAP3_high_(230512_SH_Mm_P2S525_Y3H_0506) |
| GSM7511208 | RNAPol2-Ser525_YAP3_low_(230512_SH_Mm_P2S525_Y3l_0506) |
| GSM7511209 | RNAPol2-Ser525_cholangiocacinoma_C3_(230612_RP_Mm_20230526_FFPE_O652_C3) |
| GSM7511210 | RNAPol2-Ser525_cholangiocacinoma_D3_(230612_RP_Mm_20230526_FFPE_O652_D3) |
| GSM7511211 | RNAPol2-Ser525_cholangiocacinoma_F1_(230612_RP_Mm_20230524_FFPE_O651_F1) |
| GSM7511212 | RNAPol2-Ser525_cholangiocacinoma_G1_(230612_RP_Mm_20230524_FFPE_O651_G1) |
| GSM7511213 | RNAPol2-Ser525_cholangiocacinoma_G3_(230612_RP_Mm_20230526_FFPE_O652_G3) |
| GSM7511214 | RNAPol2-Ser525_cholangiocacinoma_H1_(230612_RP_Mm_20230524_FFPE_O651_H1) |
| GSM7511215 | RNAPol2-Ser525_cholangiocacinoma_H3_(230612_RP_Mm_20230526_FFPE_O652_H3) |
| GSM7511216 | RNAPol2-Ser525_liver_A10_(230612_RP_Mm_20230520_FFPE_6541_A10) |
| GSM7511217 | RNAPol2-Ser525_liver_A12_(230612_RP_Mm_20230520_FFPE_6541_A12) |
| GSM7511218 | RNAPol2-Ser525_liver_B10_(230612_RP_Mm_20230520_FFPE_6541_B10) |
| GSM7511219 | RNAPol2-Ser525_liver_B12_(230612_RP_Mm_20230520_FFPE_6541_B12) |
| GSM7511220 | RNAPol2-Ser525_liver_C10_(230612_RP_Mm_20230520_FFPE_6541_C10) |
| GSM7511221 | RNAPol2-Ser525_liver_C12_(230612_RP_Mm_20230520_FFPE_6541_C12) |
| GSM7511222 | RNAPol2-Ser5_Normal_brain_1_(230410_SH_Mm_P2S5_N1_0330) |
| GSM7511223 | RNAPol2-Ser5_Normal_brain_1_high_(230512_SH_Mm_P2S5_N1H_0506) |
| GSM7511224 | RNAPol2-Ser5_Normal_brain_2_(230503_SH_Mm_P2S5_N95-2_0429) |
| GSM7511225 | RNAPol2-Ser5_Normal_brain_2_high_(230512_SH_Mm_P2S5_N2H_0506) |
| GSM7511226 | RNAPol2-Ser5_Normal_brain_2_low_(230512_SH_Mm_P2S5_N2l_0506) |
| GSM7511227 | RNAPol2-Ser5_Normal_brain_3_(230503_SH_Mm_P2S5_N95-12_0429) |
| GSM7511228 | RNAPol2-Ser5_Normal_brain_3_high_(230512_SH_Mm_P2S5_N3H_0506) |
| GSM7511229 | RNAPol2-Ser5_Normal_brain_3_low_(230512_SH_Mm_P2S5_N3l_0506) |
| GSM7511230 | RNAPol2-Ser5_Normal_brain_4_(230503_SH_Mm_P2S5_N90-2_0429) |
| GSM7511231 | RNAPol2-Ser5_Normal_brain_5_(230503_SH_Mm_P2S5_N85-4_0429) |
| GSM7511232 | RNAPol2-Ser5_Normal_brain_6_(230503_SH_Mm_P2S5_N85-12_0429) |
| GSM7511233 | RNAPol2-Ser5_Normal_brain_7_(230503_SH_Mm_P2S5_N80-4_0429) |
| GSM7511234 | RNAPol2-Ser5_Normal_brain_8_(230503_SH_Mm_P2S5_N80-12_0429) |
| GSM7511235 | RNAPol2-Ser5_PDGFB_1_(230410_SH_Mm_P2S5_P_0330) |
| GSM7511236 | RNAPol2-Ser5_PDGFB_4_(230428_SH_Mm_P2S5_P80_0422) |
| GSM7511237 | RNAPol2-Ser5_PDGFB_5_(230428_SH_Mm_P2S5_P85_0422) |
| GSM7511238 | RNAPol2-Ser5_PDGFB_high_(230512_SH_Mm_P2S5_PH_0506) |
| GSM7511239 | RNAPol2-Ser5_PDGFB_low_(230512_SH_Mm_P2S5_Pl_0506) |
| GSM7511240 | RNAPol2-Ser5_RELA_3_(230321_SH_Mm_P2S5_RS_0319) |
| GSM7511241 | RNAPol2-Ser5_RELA_high_(230512_SH_Mm_P2S5_RH_0506) |
| GSM7511242 | RNAPol2-Ser5_RELA_low_(230512_SH_Mm_P2S5_Rl_0506) |
| GSM7511243 | RNAPol2-Ser5_Ser25_Normal_brain_1_(230410_SH_Mm_P2S525_N1_0330) |
| GSM7511244 | RNAPol2-Ser5_Ser25_PDGFB_1_(230410_SH_Mm_P2S525_P_0330) |
| GSM7511245 | RNAPol2-Ser5_Ser25_RELA_1_(230404_SH_Mm_P2S525_R_0330) |
| GSM7511246 | RNAPol2-Ser5_Ser25_YAP1_1_(230410_SH_Mm_P2S525_Y1_0330) |
| GSM7511247 | RNAPol2-Ser5_Ser25_YAP3_1_(230410_SH_Mm_P2S525_Y3_0330) |
| GSM7511248 | RNAPol2-Ser5_YAP1_1_(230404_SH_Mm_P2S5_Y1_0330) |
| GSM7511249 | RNAPol2-Ser5_YAP1_2_(230503_SH_Mm_P2S5_Y95-2_0429) |
| GSM7511250 | RNAPol2-Ser5_YAP1_3_(230503_SH_Mm_P2S5_Y90-2_04295) |
| GSM7511251 | RNAPol2-Ser5_YAP1_4_(230503_SH_Mm_P2S5_Y85-4_0429) |
| GSM7511252 | RNAPol2-Ser5_YAP1_5_(230503_SH_Mm_P2S5_Y85-12_0429) |
| GSM7511253 | RNAPol2-Ser5_YAP1_6_(230503_SH_Mm_P2S5_Y80-12_0429) |
| GSM7511254 | RNAPol2-Ser5_YAP1_high_(230512_SH_Mm_P2S5_Y1H_0506) |
| GSM7511255 | RNAPol2-Ser5_YAP1_low_(230512_SH_Mm_P2S5_Y1l_0506) |
| GSM7511256 | RNAPol2-Ser5_YAP2_1_(230404_SH_Mm_P2S5_Y2_0330) |
| GSM7511257 | RNAPol2-Ser5_YAP2_high_(230512_SH_Mm_P2S5_Y2H_0506) |
| GSM7511258 | RNAPol2-Ser5_YAP2_low_(230512_SH_Mm_P2S5_Y2l_0506) |
| GSM7511259 | RNAPol2-Ser5_YAP3_1_(230410_SH_Mm_P2S5_Y3_0330) |
| GSM7511260 | RNAPol2-Ser5_YAP3_high_(230512_SH_Mm_P2S5_Y3H_0506) |
| GSM7511261 | RNAPol2-Ser5_YAP3_low_(230512_SH_Mm_P2S5_Y3l_0506) |
| GSM7511262 | Triton_65_K27me3_(221206_SH_Mm_K27m3_FF_TX65_1204) |

| | |
|---|---|
| GSM7511263 | Triton_65_P2S5p_(221206_SH_Mm_P2S5p_FF_TX65_1204) |
| GSM7511264 | Triton_70_P2S5p_(221206_SH_Mm_P2S5p_FF_TX70_1204) |
| GSM7511265 | Triton_75_K27me3_(221206_SH_Mm_K27m3_FF_TX75_1204) |
| GSM7511266 | Triton_75_P2S5p_(221206_SH_Mm_P2S5p_FF_TX75_1204) |
| GSM7511267 | Triton_79_K27me3_(221206_SH_Mm_K27m3_FF_TX70_1204) |
| GSM7511268 | Triton_80_K27me3_(221206_SH_Mm_K27m3_FF_TX80_1204) |
| GSM7511269 | Triton_80_P2S5p_(221206_SH_Mm_P2S5p_FF_TX80_1204) |
| GSM7511270 | Triton_85_K27me3_(221206_SH_Mm_K27m3_FF_TX85_1204) |
| GSM7511271 | Triton_85_P2S5p_(221206_SH_Mm_P2S5p_FF_TX85_1204) |
| GSM7511272 | Triton_90_K27me3_(221206_SH_Mm_K27m3_FF_TX90_1204) |
| GSM7511273 | Triton_90_P2S5p_(221206_SH_Mm_P2S5p_FF_TX90_1204) |
| GSM7511274 | Triton_95_K27me3_(221206_SH_Mm_K27m3_FF_TX95_1204) |
| GSM7511275 | Triton_95_P2S5p_(221206_SH_Mm_P2S5p_FF_TX95_1204) |
| GSM7511276 | H1_H3K4me3_whole-cell_(230421_SH_Hs_K4m3_H1_0405) |
| GSM7511277 | K562_H3K4me3_40k_whole-cell_(230421_SH_Hs_K4m3_Kl_0405) |
| GSM7511278 | K562_H3K4me3_100k_whole-cell_(230421_SH_Hs_K4m3_Kh_0405) |
| ------------------------------------------------------------------------ | |
| GSM7746188 | RNAPol2-Ser25_Normal_brain_Biomag-amine_low_(230801_SH_Mm_P2S25_Nbl_0727) |
| GSM7746189 | RNAPol2-Ser25_Normal_brain_onslide_1_(230801_SH_Mm_P2S25s_N1_0727) |
| GSM7746190 | RNAPol2-Ser25_Normal_brain_onslide_2_(230801_SH_Mm_P2S25s_N2_0727) |
| GSM7746191 | RNAPol2-Ser25_Normal_brain_onslide_3_(230801_SH_Mm_P2S25s_N3_0727) |
| GSM7746192 | RNAPol2-Ser25_Normal_brain_onslide_4_(230801_SH_Mm_P2S25s_N4_0727) |
| GSM7746193 | RNAPol2-Ser25_YAP1_Biomag-amine_low_(230801_SH_Mm_P2S25_Ybl_0727) |
| GSM7746194 | RNAPol2-Ser25_YAP1_Glutathione_high_(230801_SH_Mm_P2S25_Yph_0727) |
| GSM7746195 | RNAPol2-Ser25_YAP1_Glutathione_low_(230801_SH_Mm_P2S25_Ypl_0727) |
| GSM7746196 | RNAPol2-Ser25_YAP1_onslide_1_(230801_SH_Mm_P2S25s_Y1_0727) |
| GSM7746197 | RNAPol2-Ser25_YAP1_onslide_2_(230801_SH_Mm_P2S25s_Y2_0727) |
| GSM7746198 | RNAPol2-Ser25_YAP1_onslide_3_(230801_SH_Mm_P2S25s_Y3_0727) |
| GSM7746199 | RNAPol2-Ser25_YAP1_onslide_4_(230801_SH_Mm_P2S25s_Y4_0727) |
| GSM7746200 | RNAPol2-Ser5_Normal_brain_Biomag-amine_low_(230801_SH_Mm_P2S5_Nbl_0727) |
| GSM7746201 | RNAPol2-Ser5_Normal_brain_onslide_1_(230801_SH_Mm_P2S5s_N1_0722) |
| GSM7746202 | RNAPol2-Ser5_Normal_brain_onslide_10_(230725_SH_Mm_P2S5_N8_0720) |
| GSM7746203 | RNAPol2-Ser5_Normal_brain_onslide_2_(230801_SH_Mm_P2S5s_N2_0722) |
| GSM7746204 | RNAPol2-Ser5_Normal_brain_onslide_3_(230801_SH_Mm_P2S5s_N3_0722) |
| GSM7746205 | RNAPol2-Ser5_Normal_brain_onslide_4_(230801_SH_Mm_P2S5_N1_0720) |
| GSM7746206 | RNAPol2-Ser5_Normal_brain_onslide_5_(230801_SH_Mm_P2S5_N2_0720) |
| GSM7746207 | RNAPol2-Ser5_Normal_brain_onslide_6_(230801_SH_Mm_P2S5_N3_0720) |
| GSM7746208 | RNAPol2-Ser5_Normal_brain_onslide_7_(230801_SH_Mm_P2S5_N5_0720) |
| GSM7746209 | RNAPol2-Ser5_Normal_brain_onslide_8_(230801_SH_Mm_P2S5_N6_0720) |
| GSM7746210 | RNAPol2-Ser5_Normal_brain_onslide_9_(230801_SH_Mm_P2S5_N7_0720) |
| GSM7746211 | RNAPol2-Ser5_PDGFB_onslide_10_(230801_SH_Mm_P2S5_P1_0720) |
| GSM7746212 | RNAPol2-Ser5_PDGFB_onslide_30_(230801_SH_Mm_P2S5_P3_0720) |
| GSM7746213 | RNAPol2-Ser5_PDGFB_onslide_40_(230801_SH_Mm_P2S5_P4_0720) |
| GSM7746214 | RNAPol2-Ser5_PDGFB_onslide_50_(230801_SH_Mm_P2S5_P5_0720) |
| GSM7746215 | RNAPol2-Ser5_PDGFB_onslide_60_(230725_SH_Mm_P2S5_P6_0720) |
| GSM7746216 | RNAPol2-Ser5_PDGFB_onslide_70_(230801_SH_Mm_P2S5_P7_0720) |
| GSM7746217 | RNAPol2-Ser5_PDGFB_onslide_80_(230801_SH_Mm_P2S5_P8_0720) |
| GSM7746218 | RNAPol2-Ser5_RELA_onslide_12_(230801_SH_Mm_P2S5s_R1_0722) |
| GSM7746219 | RNAPol2-Ser5_RELA_onslide_17_(230801_SH_Mm_P2S5s_R1_0727) |
| GSM7746220 | RNAPol2-Ser5_RELA_onslide_22_(230801_SH_Mm_P2S5s_R2_0722) |
| GSM7746221 | RNAPol2-Ser5_RELA_onslide_27_(230801_SH_Mm_P2S5s_R2_0727) |
| GSM7746222 | RNAPol2-Ser5_RELA_onslide_32_(230801_SH_Mm_P2S5s_R3_0722) |
| GSM7746223 | RNAPol2-Ser5_RELA_onslide_37_(230801_SH_Mm_P2S5s_R3_0727) |
| GSM7746224 | RNAPol2-Ser5_RELA_onslide_42_(230801_SH_Mm_P2S5s_R4_0722) |
| GSM7746225 | RNAPol2-Ser5_RELA_onslide_47_(230801_SH_Mm_P2S5s_R4_0727) |
| GSM7746226 | RNAPol2-Ser5_RELA_onslide_52_(230801_SH_Mm_P2S5s_R5_0722) |
| GSM7746227 | RNAPol2-Ser5_RELA_onslide_57_(230801_SH_Mm_P2S5s_R5_0727) |
| GSM7746228 | RNAPol2-Ser5_RELA_onslide_5micron27_(230801_SH_Mm_P2S5s_5R2_0727) |
| GSM7746229 | RNAPol2-Ser5_RELA_onslide_5micron37_(230801_SH_Mm_P2S5s_5R3_0727) |
| GSM7746230 | RNAPol2-Ser5_RELA_onslide_5micron47_(230801_SH_Mm_P2S5s_5R4_0727) |
| GSM7746231 | RNAPol2-Ser5_RELA_onslide_67_(230801_SH_Mm_P2S5s_R6_0727) |
| GSM7746232 | RNAPol2-Ser5_YAP1_Biomag-amine_low_(230801_SH_Mm_P2S5_Ybl_0727) |
| GSM7746233 | RNAPol2-Ser5_YAP1_Glutathione_high_(230801_SH_Mm_P2S5_Yph_0727) |
| GSM7746234 | RNAPol2-Ser5_YAP1_Glutathione_low_(230801_SH_Mm_P2S5_Ypl_0727) |

Genome browser session
(e.g. UCSC)

No longer applicable.

## Methodology

Replicates

At least two replicates were used for Voom/Limma analysis. All replicates were successful

Sequencing depth

All Experiments were paired-end. Sequencing depths are reported in Supplementary Data 1.

| | |
|---|---|
| Antibodies | All antibodies and sources are provided in the Methods section. |
| Peak calling parameters | MACS2 version 2.2.9.1 with parameters callpeak -t <bed-file-of-mapped-fragments> -f BEDPE --keep-dup all -p 1e-5 |
| Data quality | Data quality assessment is the topic of this manuscript, and is reported. |
| Software | https://github.com/Henikoff/FFPE |
